# Adaptation of finnish diabetes risk score for screening undiagnosed diabetes and hyperglycemia in Chinese adults

**DOI:** 10.1371/journal.pone.0326914

**Published:** 2025-07-07

**Authors:** Jian Zhang, Jiating Wang, Fang Huang, Vanessa Caroline Campos, Hao Huang, Andreas Rytz, Yumeng Li, Wei Hu, Christian Darimont, Kai Yu, Yu-ming Chen

**Affiliations:** 1 Nestlé Institute of Health Sciences, Nestlé Research, Beijing, China; 2 Department of Epidemiology, Guangdong Provincial Key Laboratory of Food, Nutrition and Health, School of Public Health, Sun Yat-sen University, Guangzhou, China; 3 Nestlé Institute of Health Sciences, Nestlé Research, Lausanne, Switzerland; Tecnologico de Monterrey, MEXICO

## Abstract

**Objective:**

China has the largest population with diabetes globally, with over half of the cases going undiagnosed, highlighting the need for improved screening efforts. This study aimed to adapt the Finnish Diabetes Risk Score (FINDRSC), a widely used tool for assessing diabetes risk without relying on clinical indicators, for screening undiagnosed hyperglycemia and diabetes among Chinese adults.

**Methods:**

Data from the China Health and Nutrition Survey (CHNS), collected in the 2009 wave, were utilized as the training data (n = 7277), and data from the Guangzhou Nutrition and Health Study (GNHS, n = 2970), conducted in the years 2011–2014, were used for validation. Diabetes was defined as fasting plasma glucose (FPG) ≥ 7.0 mmol/L and/or glycated hemoglobin A1c (HbA1c) ≥ 6.5%. Hyperglycemia was defined as FPG ≥ 5.6 mmol/L and/or HbA1c ≥ 5.7%. Predictors in the original FINDRISC model were adjusted according to local standards and guidelines to develop the Modified Chinese screening model (ModChinese). Coefficients and scores of the ModChinese model were estimated using logistic regression. Area under the receiver operating characteristic curve (AUC) was calculated to evaluate model performance.

**Results:**

The prevalence of undiagnosed diabetes and prediabetes was 8.6% and 40.1% in CHNS, and 3.1% and 27.9% in GNHS, respectively. The ModChinese demonstrated superior performance compared to the original FINDRISC, with higher AUC values for detecting both diabetes (0.707 vs. 0.681, p = 0.001) and hyperglycemia (0.680 vs. 0.661, p < 0.001) in the CHNS. Similar improvements were observed in the GNHS, where the ModChinese achieved AUC values of 0.663 for diabetes and 0.606 for hyperglycemia, compared to FINDRISC’s 0.622 and 0.593, respectively. Compared with the original FINDRISC, the ModChinese model showed improved sensitivity and specificity for screening undiagnosed diabetes and enhanced sensitivity for hyperglycemia screening in both training and validation datasets.

**Conclusion:**

The ModChinese model is a simple and effective screening tool for identifying undiagnosed diabetes and hyperglycemia in Chinese adults.

## 1. Introduction

China has the largest population with diabetes in the world [1]. In 2018, it was estimated that 12.4% of Chinese adults aged 18 years or older had diabetes, which was an increase from 10.9% in 2013 [2,3]. Additionally, nearly 40% of Chinese adults have been reported to have prediabetes [3], putting them at high risk of developing diabetes during their lifetime [4]. As demographics change in China, shifting towards an ageing population [5] the number of people affected by diabetes is expected to continue to rise [6]. The increased prevalence of diabetes represents an extensive burden for individuals, families, as well as the healthcare system.

Accumulated evidence from interventional studies suggests that early interventions can be effective in reducing the incidence and delaying the progression to type 2 diabetes, while reducing associated complications [7,8]. Therefore, identifying individuals in the early stages of diabetes or prediabetes and providing timely interventions is an effective public health strategy for addressing the challenges posed by chronic disease. However, current estimates indicate that over 60% of Chinese adults with diabetes are unaware of their condition [2,3], highlighting the need for improved screening and diagnosis efforts.

To enable a more cost-effective approach in a non-clinical environment, as a screening method before confirmatory testing, researchers across the globe have developed screening tools [9–14]. These tools are recommended by international diabetes guidelines as a simple method to assist healthcare providers in making decisions regarding further diagnostic testing and interventions [15]. The China Diabetes Risk Score (CDRS), developed in 2013 [16,17], is a screening tool designed to identify undiagnosed type 2 diabetes by considering factors such as age, systolic blood pressure, body mass index (BMI), waist circumference, family history of type 2 diabetes and gender. Although the CDRS can be considered an easy-to-use tool, the inclusion of systolic blood pressure makes it less suitable for widespread applications, particularly for people living in remote areas where access to blood pressure monitoring may be limited [16]. In addition, the CDRS does not incorporate lifestyle factors, which are important components of diabetes screening tools utilized in various populations [10–13]. The inclusion of lifestyle factors is important not only because of their roles in the development of diabetes, but also because they help to raise awareness of which factors are modifiable.

The Finnish Diabetes Risk Score (FINDRISC), developed in 2003 by a team of researchers from Finland, is a simple-to-use tool designed to identify individuals at higher risk of developing type 2 diabetes [9]. The tool is non-invasive and low-cost, making it a suitable option for population-level screening in different settings. Since its development, FINDRISC has been widely used and validated across several populations and has been found to be an effective tool for identifying individuals who are at high risk of developing diabetes [18–21]. FINDRISC was first introduced to China in 2007 [22] and has since been applied in several Chinese datasets [23–25]. While these studies have adjusted the anthropometric components of FINDRISC to align with Chinese standards, they have used the original score points. However, genetic, environmental, and lifestyle differences across populations, as well as variations in the distribution of diabetes risk factors, can influence the roles of specific risk factors in diabetes development [26,27]. This highlights the need to adjust the weight of components in diabetes screening tools to accurately reflect their contribution to disease risk in a specific population. To address this, the aim of this study is to develop a new model by adjusting the FINDRISC components and re-estimating the risk scores. The goal is to develop a model that accurately reflects the risk factors for diabetes in a representative sample of the Chinese population and to test its performance.

## 2. Materials and methods

### 2.1 Study population

#### 2.1.1 Training data.

**Fig 1:**
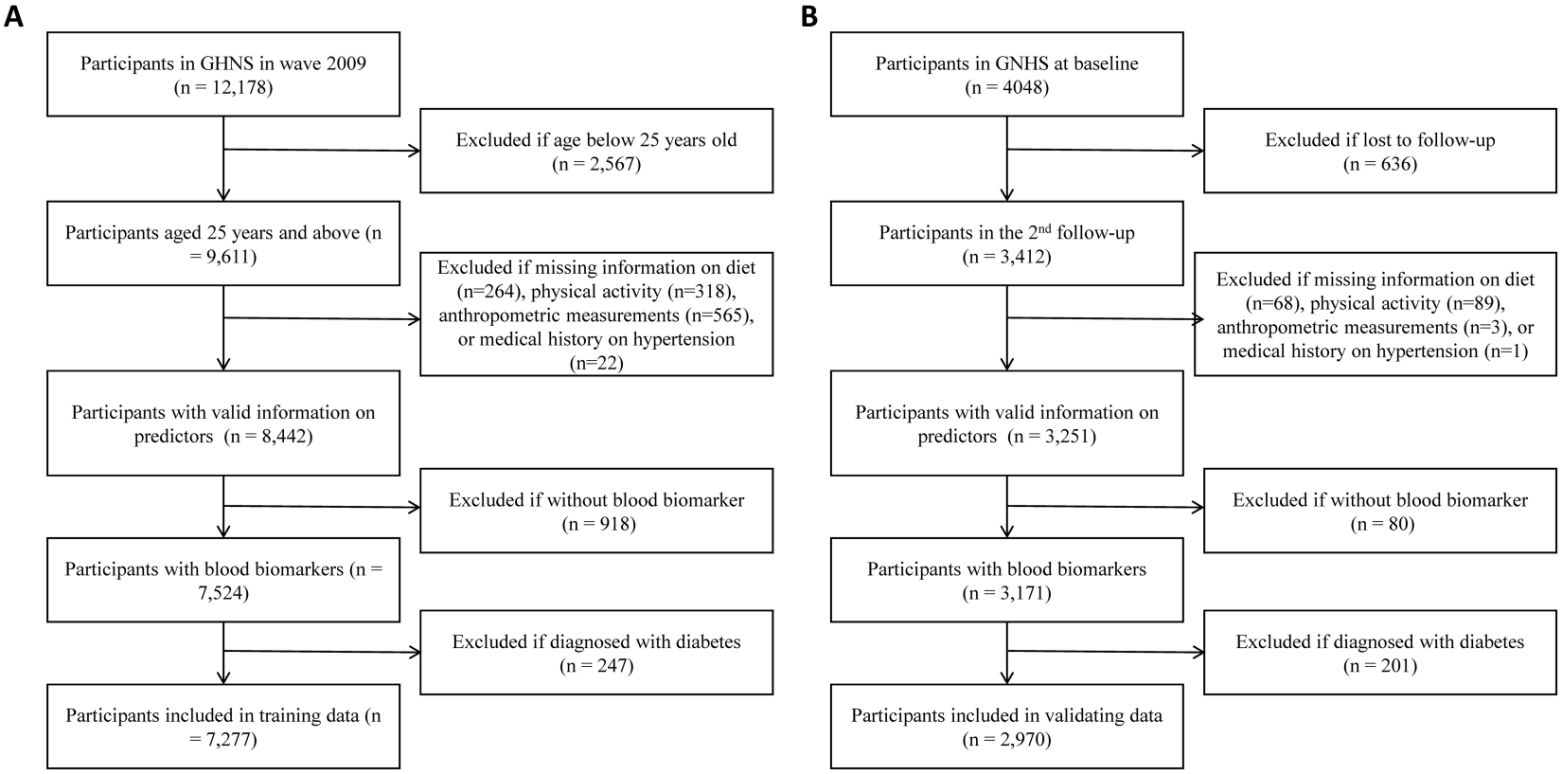
Flow chart of study participants (A) China Health and Nutrition Survey (B) Guangzhou Nutrition and Health Study.

The training dataset was derived from the China Health and Nutrition Survey (CHNS). Details about the CHNS have been published elsewhere [28,29]. In brief, the CHNS is a national cohort study conducted by the Carolina Population Center at the University of North Carolina at Chapel Hill and the National Institute for Nutrition and Health at the Chinese Center for Disease Control and Prevention. The study was designed to investigate the nutritional and health status of Chinese individuals. The CHNS was approved by the Institutional Review Committees of the University of North Carolina at Chapel Hill and the Chinese Center for Disease Control and Prevention (No. 201524) [29]. Written informed consent was obtained from all participants. The data utilized for our analysis were collected in the 2009 wave, which included participants from nine geographically diverse areas in China, covering all age groups. We excluded participants aged below 25 years (n = 2567), those who reported a prior diagnosis of diabetes (n = 247), and those who had missing information on key variables (n = 2087). The final analysis included 7,277 participants (Fig 1A).

#### 2.1.2 Validation data.

The Guangzhou Nutrition and Health Study (GNHS) is a community-based cohort study in southern China conducted by the School of Public Health, Sun Yat-sen University [30,31]. The study was designed to assess the dietary, environmental, and genetic determinants of cardiometabolic outcomes and osteoporosis. Between 2008 and 2013, a total of 4,048 participants aged between 45 and 70 years who lived in Guangzhou city for at least 5 years were enrolled in the GNHS. The GNHS was approved by the Medical Ethics Committee of Sun Yat-Sen University and registered at clinicaltrials.gov (NCT03179657). Written informed consent was obtained from all participants. Since glycated hemoglobin A1c (HbA1c) was not measured in the baseline survey, data collected in the second round of the survey (2011–2014) were used in the current analysis. Participants who were lost to follow-up (n = 636), had previously diagnosed diabetes (n = 201), or had missing information on key variables (n = 241) were excluded. The final analysis included 2,970 participants (Fig 1B).

The CHNS has a much broader scope in terms of geographical coverage and age than the GNHS, and for this reason, the CHNS was used for training and the GNHS for validation purposes in the current study.

### 2.2 Data collection and anthropometric measurements

Both the CHNS and GNHS collected information on age and sex. Trained interviewers measured participants’ waist circumference, weight, and height. BMI was calculated as weight (kg)/(height(m)^2^). Participants self-reported their history of hypertension and the use of anti-hypertensive medication.

Physical activity (PA) level was assessed using different sets of questions in the CHNS and GNHS; however, both covered a wide variety of daily-life and work-related activities [28,32]. In the current study, PA was estimated by summing the time spent on work-related activities (moderate and intense activities), commuting (walking and cycling), and exercise (all types of activities surveyed). For records with missing data in either the commute or workout section, the missing values were imputed as zero. However, if a record had two or more missing items in the commute and workout sections, the PA was treated as missing.

In the CHNS, dietary intake was assessed using a 24-hour dietary record for three consecutive days along with a household food inventory. Daily vegetable intake was calculated as mean intake across the survey days. Participants who reported consuming vegetables, fruits, and/or berries on all survey days were categorized as daily consumers. In the GNHS, habitual dietary intakes were estimated using a validated food frequency questionnaire (FFQ) [33], which covered food intake over the previous 12 months. The dietary intake was calculated based on the Chinese Food Composition Table [34]. Daily average vegetable intake was estimated, and participants who reported consuming any vegetables, fruits, and/or berries in a daily record item of the FFQ were considered daily consumers.

In the CHNS, histories of diabetes were surveyed with the question “Has a doctor ever told you that you suffer from diabetes?”. Previously diagnosed diabetes and prediabetes in the GNHS were assessed using FPG and HbA1c and the question “Have you ever been diagnosed with diabetes in a hospital?” in previous visits and the current visit with the question. Participants with confirmed diabetes were excluded from the analysis.

### 2.3 Diagnosis of diabetes, prediabetes, and hyperglycemia

In both the CHNS and GNHS, fasting venous blood was collected by medical staff. Detailed information regarding blood sample collection, storage, and measurement (including fasting plasma glucose [FPG] and HbA1c) can be found in the respective publications [29,35].

Glycemic status was defined according to the American Diabetes Association 2021 criteria [12], which have been widely used in China [2,3]. Participants with FPG ≥ 7.0 mmol/L and/or HbA1c ≥ 6.5% were classified as having diabetes; those with FPG levels between 5.6 mmol/L and 6.9 mmol/L and/or HbA1c levels between 5.7% and 6.4% were classified as having prediabetes; and the remaining participants were considered normoglycemic. Diabetes and prediabetes were collectively referred to as “hyperglycemia”. The 2-h plasma glucose level was not included in our analysis due to its absence from both datasets.

### 2.4 Adaption of FINDRISC model

To improve the performance of the FINDRISC tool in Chinese adults, we adapted the original FINDRISC model, creating the ModChinese model. Details of the comparison between the FINDRISC model and the ModChinese model can be found in S1 Table. Several adaptations were made to the original model. First, the cutoffs for BMI and waist circumference were replaced with standards specifically tailored for Chinese individuals [36]. Second, as vegetables are an essential part of the Chinese diet, the dietary information was adjusted to better reflect local eating habits. Specifically, daily vegetable consumption was measured rather than the combined intake of vegetables, fruits, and berries. The daily vegetable consumption threshold was set at 300 grams or more, in line with the daily recommendation from dietary guidelines for the Chinese population [37]. Third, sex was included in the ModChinese model, as a previous study has shown that it could improve the performance of the tool [38]. Fourth, we expanded the age range by adding a younger age group (25–34 years) to the analysis. Fifth, we replaced “*use of blood pressure medication*” with “*history of hypertension*”. Finally, the ModChinese model does not include variables related to the history of high blood glucose or family history of diabetes, as these data were not available in the CHNS.

### 2.5 Statistical methodology

General characteristics of the participants were presented as mean ± standard deviation (SD) for continuous variables and as percentages for categorical variables. Differences in continuous variables across groups were compared using ANOVA, while for categorical variables, Chi-square tests were used.

#### 2.5.1 Estimation of ModChinese model.

The original FINDRISC model began with a concise version that incorporated age, BMI, waist circumference, use of blood pressure medication, and history of high blood glucose [9]. Subsequently, physical activity and dietary intake data were added to the model, stressing the importance of lifestyle factors in the prevention of diabetes [9]. The estimation of risk scores for the ModChinese model followed the step-by-step approach used in the original FINDRISC research [9]. The first logistic regression model was conducted with diabetes as the dependent variable and age, BMI, waist circumference, and history of hypertension as predictors. In the second model, sex was also included. The third model additionally included PA and dietary information. Risk scores for the ModChinese model were defined according to the β-coefficients of the third model (β = 0.01–0.2, score = 1; β = 0.21–0.8, score = 2; β = 0.81–1.2, score = 3; β = 1.21–2.2, score = 4; β > 2.2, score = 5) [9], using the same methodology as the original FINDRISC study.

#### 2.5.2 Performance of screening model.

The performance of the original FINDRISC and the ModChinese models in screening undiagnosed diabetes was estimated by Receiver Operating Characteristic (ROC) curve. The area under the curve (AUC) was calculated and used to show the ability of the tool to identify outcomes; differences between AUCs were tested using the DeLong method from the R package *pROC.* Since both FINDRISC and ModChinese scores were integers, round numbers that yielded the highest Youden’s index were defined as cutoffs. Sensitivity, specificity, positive and negative predictive values (PV+ and PV−), positive and negative likelihood ratios, and diagnostic odds ratios at the cutoff were calculated. Validation was conducted by applying the screening model and corresponding cut-off in the GNHS data.

Subsequently, the performance of both the original FINDRISC and the ModChinese model in identifying hyperglycemia (including prediabetes and undiagnosed diabetes) were also investigated, and the corresponding cut-offs were also determined based on the Youden’s index.

All statistical analyses were performed using R 4.2.2 (R Core Team, Vienna, Austria). Additional packages used included *pROC* [39] for construction of ROC curves and *caret* [40] for calculation of sensitivity, specificity, predictive values, likelihood ratios, and diagnostic odds ratios. All statistics were two-sided, and statistical significance was defined as p < 0.05.

## 3. Results

### 3.1 General characteristics of participants

General characteristics of the participants are presented in Table 1. In the training data, the prevalence of undiagnosed diabetes and prediabetes was 8.6% and 40.1% in the CHNS and 3.1% and 27.9% in the GHNS, respectively. Participants with diabetes or prediabetes were generally older, more likely to be male, had higher BMI and waist circumference, and had a higher prevalence of hypertension. They were more likely to be on anti-hypertensive medication. Additionally, individuals with diabetes or prediabetes consumed fewer vegetables and were more likely to be physically inactive compared to those with normoglycemia. Compared with subjects in the training data, those in the validation dataset were older and had a lower prevalence of diabetes and prediabetes.

**Table 1:** Characteristics of participants in the training sample according to glycemia^1^.

Variables	China Health and Nutrition Survey				Guangzhou Nutrition and Health Study			
	Normoglycemia	Hyperglycemia^2^		*P*-value^3^	Normoglycemia	Hyperglycemia^2^		*P*-value^3^
		Prediabetes^2^	Diabetes^2^			Prediabetes	Diabetes	
n	3,736	2,918	623		2,030	848	92	
Age (years)	48.6(13.6)	54.5(13.1)	57.8(12.7)	<0.001	60.6(5.98)	61.1(5.6)	61.8(6.2)	0.027
Men (%)	44.7	47.2	50.2	0.013	31.8	31.5	31.5	0.988
Waist circumference (cm)	80.4(9.6)	84.7(9.8)	88.9(10.5)	<0.001	83.8(8.4)	86.5(8.6)	89.2(9.5)	<0.001
Body mass index (kg/m^2^)	22.7(3.1)	23.9(3.4)	25.1(3.8)	<0.001	23.1(2.9)	24.3(3.1)	25.6(3.6)	<0.001
Hypertension (%)	8.4	16.5	25.2	<0.001	24.3	33	42.4	<0.001
Anti-hypertension medication (%)	6.2	13.5	19.9	<0.001	21.6	29.2	35.9	<0.001
Fail to intake fruits and/or vegetables everyday (%)^3^	4.7	6.5	7.2	0.001	45.7	44.2	43.5	0.735
Average vegetable intake < 300g/d (%)^4^	44.7	49.6	49.9	<0.001	46.0	42.2	57.6	0.010
Daily work or leisure-related PA < 0.5h (%)	47.8	54.1	60.2	<0.001	0.6	0.8	0	0.569

^1^ Continuous variables were presented as means and SDs, and categorical variables were presented as percentages.

^2^ Diabetes was defined as having fasting plasma glucose (FPG) ≥ 7.0 mmol/L and/or glycated hemoglobin A1c (HbA1c) ≥ 6.5%. Prediabetes was defined as FPG between 5.6–6.9 mmol/L and/or HbA1c between 5.7–6.4%. Diabetes and prediabetes were collectively referred to as ‘hyperglycemia’.

^3^ Difference across groups were compared using ANOVA for continuous variables and chi-square for categorical variables.

^4^ Dietary intake status were defined by data collected by 3d dietary recall in China Nutrition and Health Survey and 1-year food frequency questionnaire in Guangzhou Nutrition and Health Study, respectively.

### 3.2 ModChinese model

The first model showed that age, BMI, waist circumference, and history of hypertension were all significant predictors of undiagnosed diabetes in Chinese adults. Sex was also found to be a significant predictor of diabetes, but its inclusion had minimal impact on the coefficients of the other variables (second model). In the third model, daily vegetable consumption (< 300 g) and daily PA (< 0.5 h) were added. However, like the original FINDRISC model, these variables did not contribute significantly to the predictive power of the model. Nevertheless, they were retained in the ModChinese model to emphasize the importance of lifestyle factors in diabetes prevention. The risk scores of the ModChinese model were defined according to the β-coefficients of the third model, as presented in Table 2.

**Table 2:** Development of ModChinese model in China Health and Nutrition Survey (n = 7,277) for screening diabetes [β-coefficient (p-value)]^1^.

Variable	Cases/non-cases	Model 1	Model 2	Model 3/ ModChinese	Score
Age (years) ^2^					
25-34	18/759	Ref	Ref	Ref	0
35-44	85/1,527	0.73(0.006)	0.74(0.006)	0.76(0.004)	2
45-54	145/1,698	1.03(<0.001)	1.04(<0.001)	1.05(<0.001)	3
55-	375/2,670	1.49(<0.001)	1.50(<0.001)	1.47(<0.001)	4
BMI (kg/m^2^)					
<24	255/4,088	Ref	Ref	Ref	0
24-27	230/2,014	0.19(0.090)	0.17(0.090)	0.18(0.122)	1
28	138/552	0.72(<0.001)	0.71(<0.001)	0.71(<0.001)	2
Waist circumference (cm)					
Men, < 85; Women, < 80	162/3,317	Ref	Ref	Ref	0
Men, 85–95; Women, 80–90	224/2,208	0.5(<0.001)	0.54(<0.001)	0.53(<0.001)	2
Men, ≥ 96; Women, ≥ 90	237/1,129	0.89(<0.001)	0.95(<0.001)	0.93(<0.001)	3
History of hypertension					
No	466/5,858	Ref	Ref	Ref	0
Yes	157/796	0.40(<0.001)	0.40(<0.001)	0.39(<0.001)	2
Gender					
Female	310/3,609	–	Ref	Ref	0
Male	313/3,045	–	0.30(<0.001)	0.33(<0.001)	2
Vegetables < 300g/d					
No	312/3,537	–	–	Ref	0
Yes	311/3,117	–	–	0.13(0.122)	1
Physical activity < 0.5h/d					
No	248/3,288	–	–	Ref	0
Yes	375/3,366	–	–	0.16(0.084)	1

^1^ Logistic regression models were conducted with diabetes as dependent variable and components of the screening tool as independent variables. Values shown in the table were β-coefficients and p-values, unless stated otherwise. ModChinese: diabetes screening model modified from Finnish Diabetes Risk Score. Ref: reference.

^2^ Age group 55–64 and ≥65 years were combined because these two groups get the same scores.

### 3.3 Performance of original FINDRISC and ModChinese

In the training data, both original FINDRISC and ModChinese models had score ranges from 0 to 15. The ModChinese model had higher AUC (0.707) than the original FINDRISC model (AUC: 0.681) in screening for undiagnosed diabetes (Table 3 and S1 Fig). At a cut-off of 8, the ModChinese model achieved the highest Youden’s index, indicating optimal sensitivity and specificity, while the corresponding value for the FINDRISC model was 6. For hyperglycemia, the AUC of the ModChinese model was also higher than the original FINDRISC model (0.680 vs. 0.661). The cut-off of ModChinese model for screening hyperglycemia was 7, while that of the original FINDRISC model was 6, which was the same as the cutoff for screening diabetes.

**Table 3:** Performance of FINDRISC and ModChinese in screening diabetes and hyperglycemia^1^.

	AUC^2^	P-value	Cutoffs^3^	True negative	False positive	False negative	True positive	Sensitivity	Specificity	Positive predictive value	Negative predictive value	Positive likelihood ratio	Negative likelihood ratio	Diagnostic odds ratio
**China Health and Nutrition Survey**														
Original FINDRISC for diabetes	0.681	0.001	6	4059	2595	211	412	0.661	0.610	0.137	0.951	1.696	0.555	3.054
ModChinese for diabetes	0.707		8	4146	2508	198	425	0.682	0.623	0.145	0.954	1.810	0.510	3.548
Original FINDRISC for hyperglycemia	0.661	<0.001	6	2625	1111	1645	1896	0.535	0.703	0.631	0.615	1.801	0.661	2.723
ModChinese for hyperglycemia	0.680		7	2220	1516	1157	2384	0.673	0.594	0.611	0.657	1.659	0.550	3.017
**Guangzhou Nutrition and Health Study**														
Original FINDRISC for diabetes	0.622	0.122	6	1199	1679	27	65	0.707	0.417	0.037	0.978	1.213	0.703	1.726
ModChinese for diabetes	0.663		8	1519	1359	26	66	0.717	0.528	0.046	0.983	1.519	0.536	2.834
Original FINDRISC for hyperglycemia	0.593	0.164	6	920	1110	306	634	0.675	0.453	0.364	0.750	1.234	0.717	1.720
ModChinese for hyperglycemia	0.606		7	777	1253	244	696	0.740	0.383	0.357	0.761	1.199	0.679	1.767

^1^ FINDRISC: Finnish Diabetes Risk Score. ModChinese: diabetes screening model modified from Finnish Diabetes Risk Score. Diabetes was defined as having fasting plasma glucose (FPG) ≥ 7.0 mmol/L and/or glycated hemoglobin A1c (HbA1c) ≥ 6.5%. Hyperglycemia was defined as having FPG ≥ 5.6 mmol/L and/or HbA1c ≥ 5.7%.

^2^ AUC: area under the curve. Difference between AUCs (FINDRISC vs. ModChinese) were tested with the DeLong method. P-value: for the differences between AUCs derived from the ModeChines and original FINDRISC model was tested using the DeLong method

^3^ Cutoffs were defined in terms of the largest Youden’s Index.

In the validation data, the AUC values of the ModChinese model and the original FINDRISC model were 0.663 and 0.622, respectively, in screening for undiagnosed diabetes, and 0.606 and 0.593 for hyperglycemia. Using the cut-offs derived from the training data, the ModChinese model had higher sensitivity, specificity, PV+ and PV- compared to the original FINDRISC model. For hyperglycemia, the ModChinese model had higher sensitivity but lower specificity (Table 3).

## 4. Discussion

To develop a diabetes screening tool suitable for application in China, we adapted the FINDRISC into the ModChinese model. This adaptation was based on a cross-sectional, nationally representative dataset of Chinese adults, and the performance of the model was validated using a separate dataset. Our study demonstrated that the ModChinese model outperformed the original FINDRISC in identifying undiagnosed diabetes and hyperglycemia among the Chinese population.

To ensure that the tool is more suitable for the Chinese population, several adjustments were made. It has been long recognized that obesity is a key risk factor in the development of diabetes. However, evidence from observational studies suggested there are ethnic differences in obesity phenotypes and disparities in the association of obesity with diabetes [26,27]. Evidence from the UK Biobank study suggests that non-white groups, including Chinese, South Asian, and Black individuals, show a higher prevalence of diabetes at lower levels of adiposity. Applying uniform obesity standards across different ethnic groups may lead to underestimation of the disease risk [41]. Therefore, it has been recommended that different cut-offs be applied for different ethnic groups in disease screening and intervention [41]. To make the ModChinese model more appropriate for the Chinese population, we adjusted the BMI and waist circumference cut-offs, which were originally based on Western populations, to standards specifically tailored for Chinese individuals [36,42].

In the original FINDRSC model, only individuals aged above 45 years received extra points for the age component. However, recent data show an increasing prevalence of young-onset diabetes in China [43,44]. According to a national Chinese survey conducted in 2018, among those aged 30–39 years, the prevalence of diabetes and prediabetes was 6.5% and 34.2%, respectively, which represents a concerning trend [3]. To address this issue and capture the risk of diabetes in younger age groups, we added a younger age category to the ModChinese model. Our results showed that, compared to participants aged 25–34 years, those aged 35–44 years had significantly increased odds of diabetes, thereby justifying the age adjustment.

Furthermore, epidemiological evidence consistently demonstrates that men have a higher prevalence of diabetes compared to women across different ethnicities [45,46]. Although the original FINDRISC model excluded sex due to the potential impact on the coefficients of other variables [9], subsequent studies showed that adding sex can enhance performance of the model [38]. In the current study, sex was found to be a significant predictor of undiagnosed diabetes, but its inclusion had minimal impact on the scores of other variables (model 2 vs. model 1). Therefore, it was decided to retain sex as a variable in the ModChinese model.

Additionally, previous research has suggested that the application of cut-offs for daily consumption of vegetables, fruits, or berries may not work effectively in certain populations, such as in the Philippines, where high consumption rates are observed [47]. Similarly, in our study population, a high daily consumption of vegetables or fruits was reported, with over 90% of individuals in the CHNS dataset reporting daily intake of fruits and vegetables. Moreover, vegetables have been found to be more relevant to the prevention of diabetes in the Chinese population [48]. To improve the discriminative power of this variable, a new cut-off was proposed, setting the average daily vegetable consumption at greater than or equal to 300 grams. This cut-off aligns with the recommendations from the dietary guidelines for the Chinese population [37].

In contrast to previous studies, we further re-estimated the risk scores for the adapted model by adjusting the cut-off values of FINDRISC components. Our findings showed significant changes in risk scores, particularly for the age and adiposity indices. In the adapted ModChinese model, an additional point was assigned for the same age group compared to the original FINDRISC model (e.g., the score for the 45–54 age group was 1 in FINDRISC and 2 in ModChinese), and the age variable exhibited a ceiling effect, as participants in the 55–65 and >65 years age groups received similar scores. This result may be attributed to the younger age of diabetes onset observed in the Chinese population compared to their Caucasian counterparts [44], suggesting that Chinese individuals face a greater risk of diabetes at the same age, which is reflected in the adapted model. In contrast, risk scores for the top groups of adiposity indicators (BMI and waist circumference) decreased by one point. This change may be related to the lower cut-off values used in the ModChinese model compared to the original FINDRISC model (e.g., 30 kg/m2 in FINDRISC vs. 28 kg/m2 in ModChinese).

In our study, after adjustment and re-estimation, the AUC for diabetes screening improved from 0.681 to 0.708. Additionally, we observed increases in sensitivity, specificity, PV + , and PV- in both the training and validation datasets. Although the magnitude of the improvements was modest, we believe they may have a significant impact in a real-world settings. Firstly, the adjustment and re-estimation of ModChinese model make it more generalizable and reliable to the general Chinese population. The predictors and corresponding cut-offs age aligned with local guidelines, making the tool easier for healthcare professionals to interpret. This alignment enhances the tool’s relevance and ensures that it accurately reflects the risk factors for diabetes in the Chinese population. Secondly, as an easy-to-use and non-invasive tool, the ModChinese model can be implemented across multiple platforms (e.g., cellphones), enabling the early identification of individuals at risk for diabetes. Early risk identification and management can facilitate timely intervention and ultimately improve health outcomes.

Aside from using the risk score estimation method used in the original FINDRISC research [9], which converts beta-coefficients into integer-based risk scores, we also calculated probabilities directly from the logistic regression model and used these probabilities solely as predictors in the screening for diabetes. The probability-based approach was proposed to minimize information loss, however, it came at the expense of an intuitive and interpretable scoring system. Given that the performance gain was marginal, we ultimately decided to retain the original FINDRISC scoring method.

Regarding the screening for prediabetes, previous studies have demonstrated that the FINDRISC model achieves moderate performance across various populations (e.g., AUC = 0.67 in the US [49], 0.63 in Spanish [18], 0.79 in Malaysia [20], and 0.72 in Indonesia [50]). However, in our study population, the original model appeared to be suboptimal for screening hyperglycemia. Specifically, the cut-off yielding the highest Youden’s Index was the same as that for diabetes and lowering the cut-off substantially compromise specificity. To address this limitation, we applied the ModChinese score to screen for hyperglycemia and identified a cutoff of 7, which was one point lower than that used for diabetes. The better performance of this model suggests its practical potential for screening both diabetes and hyperglycemia in the Chinese population.

The major strength of the current study lies the adaptation of predictors in the FINDRISC screening tool in accordance with Chinese guidelines and the use of a large, nationally representative dataset to update the risk scores. However, several limitations should be acknowledged. First, the absence of information on family history of diabetes and history of high blood glucose in the CHNS dataset necessitated the exclusion of these variables from the analysis, which may have reduced the diagnostic accuracy when compared to scenarios where these variables are included. Nevertheless, the performance of the screening tool remained acceptable, with an AUC exceeding 0.7. Second, the diagnosis of diabetes and prediabetes was based on FPG and HbA1c levels only, and not included the 2-h post-load venous plasma glucose test, which may have led to some undiagnosed cases of diabetes. Third, the study could not distinguish between type 1 and type 2 diabetes. However, given the significantly higher prevalence of type 2 diabetes in the Chinese adults [51], it was assumed that the majority of newly detected cases with diabetes in the current study represented type 2 diabetes. Fourth, validation of the ModChinese model was conducted using data from a regional Chinese population. Future work may be needed to validate the tool’s performance in broader and more diverse populations. Lastly, the relatively low specificity and Youden’s index observed in the model may increase the likelihood of false negatives and necessitate confirmatory testing. Therefore, the score’s performance should be interpreted within the context of its intended role as a preliminary screening tool, to be followed by clinical validation.

## 5. Conclusion

The ModChinese can serve as an effective tool to enhance individuals’ awareness of their diabetes risk by identifying those at high risk for diabetes and hyperglycemia, thereby providing a foundation for early management. Its simplicity and non-invasive nature make it suitable for use in the general population, particularly in non-clinical settings. Future studies should aim to incorporate information on a history of high blood glucose and family history of diabetes, and to assess their impact on the tool’s performance. This may further improve the accuracy and effectiveness of the ModChinese screening tool in identifying individuals at risk and facilitating early intervention for diabetes and hyperglycemia.

## Supporting information

S1 Table Comparison between FINDRISC and ModChinese. (DOCX)

S1 Fig Receiver operating characteristic curves for detecting undiagnosed diabetes (a) and hyperglycemia (b) in China Health and Nutrition Survey of 2009. (DOCX)
