# Supplementary material for: Adaptation of finnish diabetes risk score for screening undiagnosed diabetes and hyperglycemia in Chinese adults
Source: PLoS One. 2025 Jul 7;20(7):e0326914. doi: 10.1371/journal.pone.0326914 (PMC12233222; doi:10.1371/journal.pone.0326914)
Supplement: S1 Table — (DOCX) [file pone.0326914.s001.docx]

**Supporting information**

**Table S1.** Comparison between FINDRISC and ModChinese^1^

|  | FINDRISC | | ModChinese | |
| --- | --- | --- | --- | --- |
| Age (years) | < 45  45 - 54  55 – 64  > 64 | | 25 - 34  35 - 44  45 - 54  ≥ 55 | |
| Body mass index (kg/m^2^) | <25  25-30  >30 | | < 24  24 - 27  ≥ 28 | |
| Waist circumference (cm) | Men  < 94  94 - 102  > 102 | Women  < 80  80- 88  > 88 | Men  < 85  85 - 94  ≥ 95 | Women  < 80  80 - 89  ≥ 90 |
| Physical activity | Time at work and/or leisure ≥ 30 min/d | | | |
| Diet | Eat vegetables, fruits or berries on daily basis | | Daily vegetable intake ≥300g | |
| Hypertension | Take medication for high blood pressure | | History of hypertension | |
| Gender | - | | Being male | |

^1^ History of high blood glucose and family history of diabetes were not included. FINDRISC: Finnish Diabetes Risk Score. ModChinese: diabetes screening model modified from FINDRISC.
