# Supplementary material for: Adaptation of finnish diabetes risk score for screening undiagnosed diabetes and hyperglycemia in Chinese adults
Source: PLoS One. 2025 Jul 7;20(7):e0326914. doi: 10.1371/journal.pone.0326914 (PMC12233222; doi:10.1371/journal.pone.0326914)
Supplement: S1 Fig — (DOCX) [file pone.0326914.s002.docx]

**Supporting information**


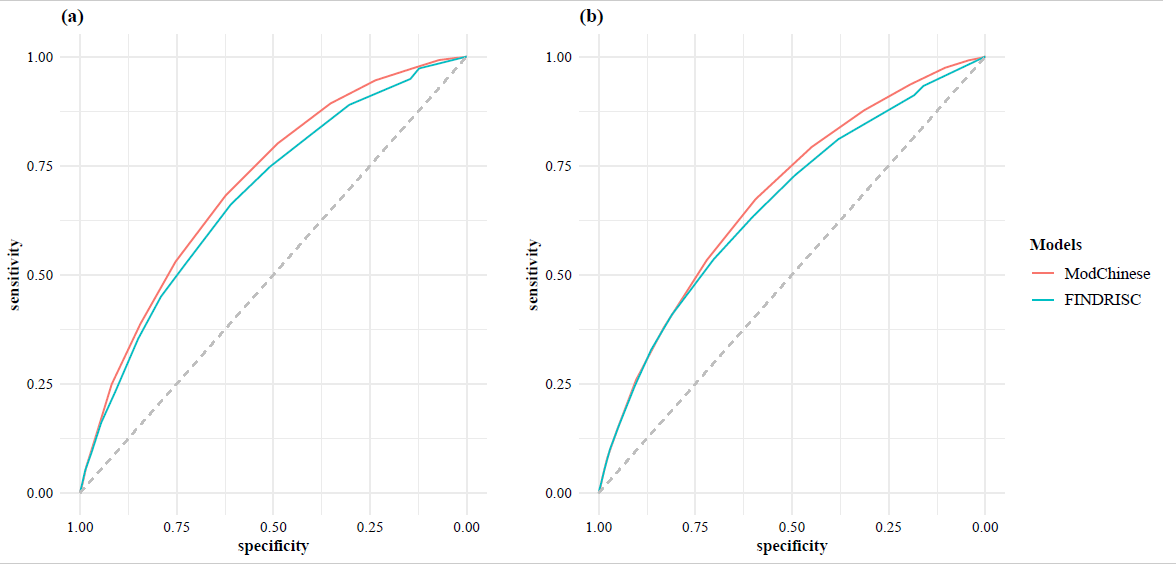


**Fig S1.** Receiver operating characteristic curves for detecting undiagnosed diabetes (a) and hyperglycemia (b) in China Health and Nutrition Survey of 2009.
